# Neuroimaging in Nonsyndromic Craniosynostosis: Key Concepts to Unlock Innovation

**DOI:** 10.3390/diagnostics14171842

**Published:** 2024-08-23

**Authors:** Camilla Russo, Ferdinando Aliberti, Ursula Pia Ferrara, Carmela Russo, Domenico Vincenzo De Gennaro, Adriana Cristofano, Anna Nastro, Domenico Cicala, Pietro Spennato, Mario Quarantelli, Marco Aiello, Andrea Soricelli, Giovanni Smaldone, Nicola Onorini, Lucia De Martino, Stefania Picariello, Stefano Parlato, Peppino Mirabelli, Lucia Quaglietta, Eugenio Maria Covelli, Giuseppe Cinalli

**Affiliations:** 1 Neuroradiology Unit, Department of Pediatric Neurosciences, Santobono-Pausilipon Children’s Hospital, 80129 Naples, Italy; 2 Cranio-Maxillo-Facial Surgery Unit, Department of Pediatric Neurosciences, Santobono-Pausilipon Children’s Hospital, 80129 Naples, Italy; 3 Pediatric Neurosurgery Unit, Department of Pediatric Neurosciences, Santobono-Pausilipon Children’s Hospital, 80129 Naples, Italygiuseppe.cinalli@gmail.com (G.C.); 4 Institute of Biostructures and Bioimaging, Italian National Research Council, 80145 Naples, Italy; 5 IRCCS SYNLAB SDN, 80143 Naples, Italygiovanni.smaldone@synlab.it (G.S.); 6 Neuro-Oncology Unit, Department of Pediatric Oncology, Santobono-Pausilipon Children’s Hospital, 80129 Naples, Italy; 7 Clinical and Translational Research Unit, Santobono-Pausilipon Children’s Hospital, 80129 Naples, Italy

**Keywords:** craniosynostosis, craniofacial surgery, neuroradiology, magnetic resonance imaging, computed tomography, blackbone MRI, arterial spin labeling, magnetic resonance angiography, perfusion MRI

## Abstract

Craniosynostoses (CRS) are caused by the premature fusion of one or more cranial sutures, with isolated nonsyndromic CRS accounting for most of the clinical manifestations. Such premature suture fusion impacts both skull and brain morphology and involves regions far beyond the immediate area of fusion. The combined use of different neuroimaging tools allows for an accurate depiction of the most prominent clinical–radiological features in nonsyndromic CRS but can also contribute to a deeper investigation of more subtle alterations in the underlying nervous tissue organization that may impact normal brain development. This review paper aims to provide a comprehensive framework for a better understanding of the present and future potential applications of neuroimaging techniques for evaluating nonsyndromic CRS, highlighting strategies for optimizing their use in clinical practice and offering an overview of the most relevant technological advancements in terms of diagnostic performance, radiation exposure, and cost-effectiveness.

## 1. Introduction

Craniosynostoses (CRS) are developmental craniofacial anomalies characterized by the premature fusion of one or more major cranial sutures; in primary CRS, the early fusion of one or more sutures is thought to be the result of a developmental error occurring during embryogenesis, while in secondary CRS, it is considered the result of external causes impacting intrauterine growth or early infancy. This condition can lead to an abnormally shaped skull; overall cranial growth reduction, sometimes resulting in increased intracranial pressure (ICP); and impaired brain development, potentially leading to neurological complications (such as developmental delays, sensory impairment, and respiratory dysfunction) [1,2]. Recent studies have shown that premature suture fusion can also impact brain morphology, involving regions beyond the immediate area of fusion. These alterations do not completely recede despite surgical correction, and children often continue to show altered brain growth patterns [3]. These data are consistent with the evidence in the literature, highlighting how putative etiologic factors encompass genetic, intrauterine, and environmental causes. Genetic mutations play a significant role, with sporadic mutations (rather than inherited patterns) considered more common in nonsyndromic cases. Mutations in genes such as FGFR2, TWIST1, and EFNB1 have been called into question, as their impairment may be responsible for premature suture ossification, abnormal osteoblast differentiation, and overactive autophagy disrupting normal bone homeostasis [4,5,6]. Environmental factors, such as maternal smoking, advanced paternal age, and the use of certain medications during pregnancy, also negatively impact normal suture fusion. Intrauterine factors, such as restricted fetal head movement due to insufficient amniotic fluid or multiple pregnancies, may also contribute [7].

In clinical practice, craniosynostoses are classified based on the involved sutures as simple (if involving one single suture) or complex (involving two or more sutures) and can be either syndromic (if associated with other anomalies) or nonsyndromic (if occurring as isolated defects). The overall incidence ranges between 1:2.000 and 1:2.500, with a slightly greater incidence in males than in females. Syndromic craniosynostoses (i.e., associated with genetic syndromes such as Apert, Pfeiffer, and Crouzon) represent approximately 15–30% of cases, whereas the majority are nonsyndromic. Unlike syndromic cases, nonsyndromic craniosynostoses generally affect a single suture. Sagittal synostosis is the most common type, accounting for 40–60% of cases, followed by coronal (approximately 20%), metopic (approximately 15%), and lambdoid synostosis (approximately 5%) [1,8].

Historically, according to Virchow’s law [9], when a suture closes early, the skull cannot grow perpendicularly to that suture and instead grows parallel to it. This principle predicts the resultant shape of the cranial deformity. Conversely, according to an alternative explanation known as the “functional matrix hypothesis” by Melvin Moss [10], as brain development normally precedes the complete ossification of the calvarium, the brain, and meninges play a primary role in the formation and maintenance of cranial suture patency, thus influencing the shape of the cranial vault and determining compensatory growth in the case of impaired suture fusion. However, studies on CRS indicate that the skull and brain are so closely interconnected that neither one predominates in the determination of the final cranial shape, which can, therefore, be considered the result of a continuous biomechanical interplay between underlying soft tissues and overlying developing bones [11].

The major types of nonsyndromic craniosynostoses include sagittal, unicoronal, bicoronal, metopic, and lambdoidal craniosynostoses (resulting in scaphocephaly, anterior plagiocephaly, brachycephaly, trigonocephaly, and posterior plagiocephaly, respectively). The treatment primarily involves surgery, aiming to correct skull deformity, allow for normal brain growth, and mitigate ICP increase. The optimal age for surgery is between 6 and 12 months. Surgical techniques range from endoscopic suturectomy to open cranial vault reconstruction, the timing of which is influenced by the pervasiveness and severity of the condition [12,13,14,15]. Complications mainly include bleeding, cerebrospinal fluid (CSF) leakage, and postoperative infections, which are more common in cases of open surgery. The need for reoperation, although not common in cases of nonsyndromic craniosynostoses, can occasionally be needed. Early diagnosis and attentive postoperative follow-up are essential for the successful treatment of these patients.

Usually, recognizable since early infancy, a nonsyndromic craniosynostosis diagnostic assessment starts from a comprehensive clinical evaluation, followed by an imaging assessment routinely based on computed tomography (CT) scans with orthogonal and volumetric reconstructions. Concerning neuroimaging tools, plain skull radiography has been almost completely abandoned as a diagnostic technique in daily clinical practice, whereas transcranial ultrasound (US) and magnetic resonance imaging (MRI) are gaining new ground as possible alternatives or complements to CT evaluation [16,17]. Indeed, CT has long been the gold standard for evaluating CRS. However, concerns about radiation exposure in pediatric patients have led to the exploration of alternative imaging techniques (above all, black-bone MRI).

The aim of this review is to outline the development of neuroimaging techniques applied to nonsyndromic CRS, examine all relevant imaging methods, and explore the role of the latest technological advancements, in terms of diagnostic performance, radiation exposure, and cost-effectiveness. This paper will also highlight the most relevant clinical–radiological presentations of nonsyndromic craniosynostoses to demonstrate the optimal use of the most common imaging techniques, with particular reference to magnetic resonance imaging (MRI) and postsurgical patient monitoring.

## 2. Major Types of Nonsyndromic Craniosynostosis

**Figure 1:**
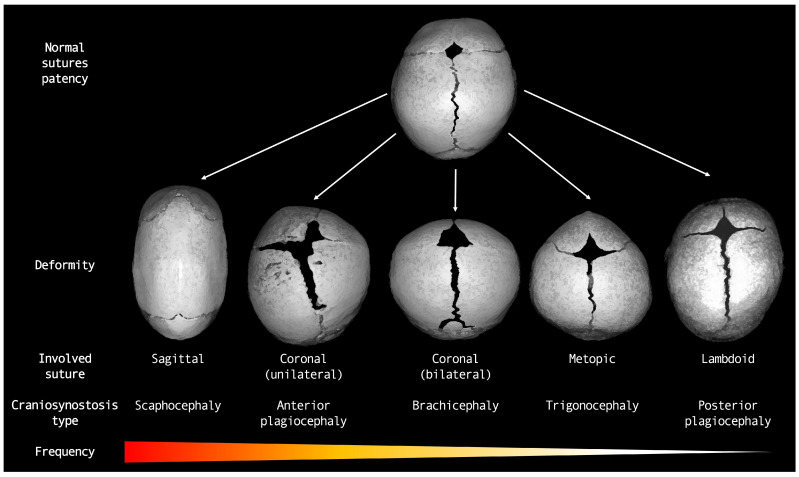
Diagram representing major types of nonsyndromic craniosynostoses.

Before describing the optimal use of neuroimaging techniques for investigating the most prominent clinical–radiological features of premature suture fusion, we provide a brief summary of the major types of nonsyndromic craniosynostoses and their key features [18] (Figure 1). The physiological timeline of cranial vault suture fusion is also shown in Table 1.

### 2.1. Sagittal Craniosynostosis (Scaphocephaly)

**Figure 2:**
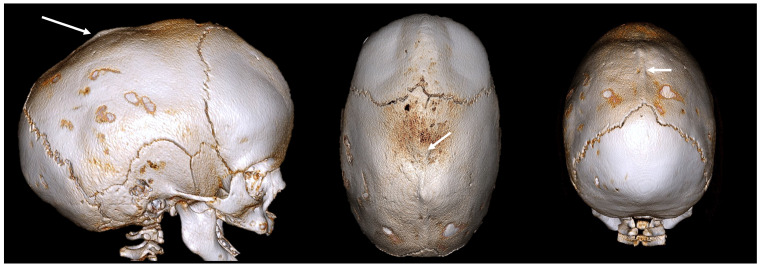
A 3D CT image of a sagittal CRS (scaphocephaly) with an elongated skull, a prominent forehead, and occipital bulging. The white arrow indicates an abnormally fused suture.

Sagittal CRS is the most common form of nonsyndromic CRS. It involves the premature fusion of the sagittal suture, which runs from the front to the back of the skull. This fusion restricts the lateral growth of the skull, resulting in a long, narrow head shape known as scaphocephaly (or dolichocephaly) (Figure 2).

The key features are:

- Elongated, boat-shaped skull;
- Prominent forehead;
- Occipital bulging;
- Possible developmental delays due to restricted brain growth.

### 2.2. Coronal Craniosynostosis

**Figure 3:**
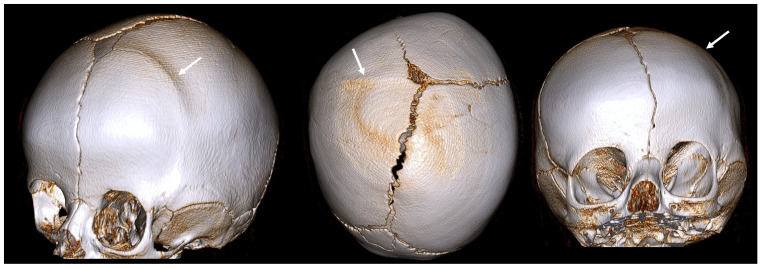
A 3D CT image of a unilateral coronal CRS (anterior plagiocephaly) with an asymmetrical forehead, a flattened forehead and brow on the affected side, an elevated eye cavity on the affected side, and a nose that deviated toward the affected side. The white arrow indicates an abnormally fused suture.

**Figure 4:**
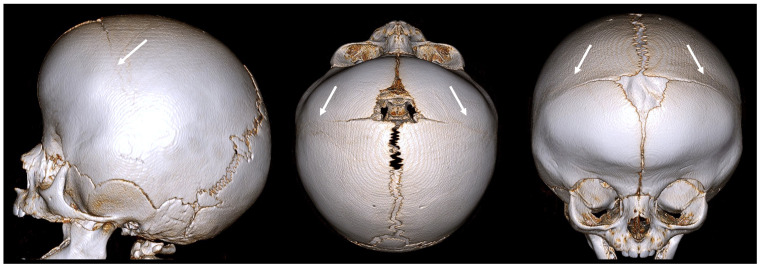
A 3D CT example of bilateral coronal CRS (brachycephaly) with symmetrical flattening of the forehead, short broad skull, and increased ICP due to restricted growth. The white arrow indicates an abnormally fused suture.

Coronal CRS affects the coronal sutures, which extend from ear to ear across the top of the skull. It can be unilateral (Figure 3) or bilateral (Figure 4).

The key features of unilateral coronal craniosynostosis (anterior plagiocephaly) are:

- asymmetrical forehead;
- flattened forehead and brow on the affected side;
- elevated eye socket on the affected side;
- nose deviated toward the affected side.

The key features of bilateral coronal craniosynostosis (brachycephaly) are:

- symmetrical flattening of the forehead;
- short, broad skull;
- increased ICP due to restricted growth.

### 2.3. Metopic Craniosynostosis (Trigonocephaly)

**Figure 5:**
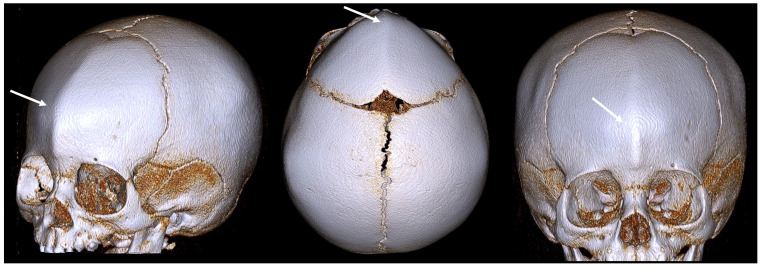
A 3D CT example of metopic CRS (trigonocephaly) with a triangular keel-shaped forehead, hypotelorism, and a midline ridge along the forehead. The white arrow indicates an abnormally fused suture.

Metopic CRS involves the premature fusion of the metopic suture, which runs from the top of the head down the middle of the forehead to the nose. This leads to a triangular forehead, a condition also known as trigonocephaly (Figure 5).

The key features are:

- triangular, keel-shaped forehead;
- closely spaced eyes (hypotolorism);
- midline ridge along the forehead;
- potential cognitive and developmental impairments.

### 2.4. Lambdoid Craniosynostosis (Posterior Plagiocephaly)

**Figure 6:**
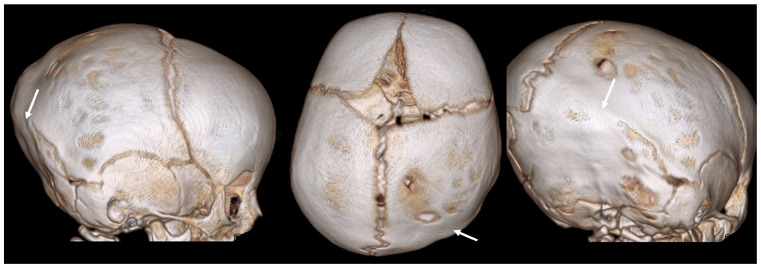
A 3D CT image of a lambdoid CRS (posterior plagiocephaly) with asymmetrical flattening of the back of the head, misalignment of the jaws, ears, and facial structures, and a tilted cranial base. The white arrow indicates an abnormally fused suture.

Lambdoid CRS is the rarest form of premature suture fusion and involves the use of lambdoid sutures at the back of the skull (Figure 6). This condition should not be confused with positional plagiocephaly, a nonsynostotic deformity caused by external pressure due to the prolonged supine position of infants during the first months of life (thus molding the head into an asymmetrical shape).

The key features are:

- asymmetrical flattening of the back of the head;
- one ear positioned higher than the other;
- tilted cranial base;
- misalignment of the jaw and facial structures.

### 2.5. Mixed and Complex Craniosynostosis

**Figure 7:**
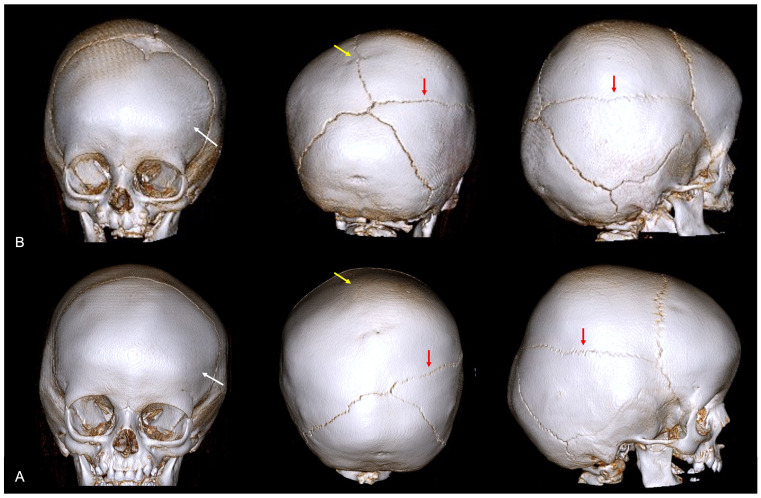
A 3D CT image of complex nonsyndromic CRS in a 6-month-old patient diagnosed with skull deformity. (**A**) Right frontal eminence asymmetry (white arrow) and minor left posterior plagiocephaly, with incipient sagittal synostosis (yellow arrow) and the presence of a right parietal accessory suture (red arrow). Subsequent 1-year follow-up examination (**B**) confirmed mild skull deformity due to complete fusion of the sagittal suture, not resulting in classic scaphocephaly due to patency of the PA suture.

Some cases involve the premature fusion of multiple major or minor sutures, leading to complex cranial shapes and increased ICP. These cases can present with a combination of features from different types of craniosynostoses and, occasionally, can be nonsyndromic (Figure 7). Genetic mutations are major determining factors in complex craniosynostoses [19].

The key features are:

- mixed deformities depending on which sutures are involved;
- severe cranial asymmetry;
- high risk of developmental delays and neurological issues;
- complex surgical planning required for correction.

## 3. Optimizing Imaging Methods: Moving beyond Ionizing Radiation

Neuroimaging techniques have advanced significantly in recent years, progressing from simple skull radiographs to sophisticated acquisition techniques that allow for volumetric visualization of the skull. Moreover, neuroimaging is also crucial for assessing the severity of the condition, identifying any coexisting abnormality, detecting possible disease-related complications early, and aiding in both surgical planning and posttreatment monitoring.

Historically, X-rays and bone scintigraphy have played significant roles in the evaluation of CRS. X-rays, widely used in the mid-20th century, provided essential images of cranial sutures, helping to identify premature fusions. However, their limited detail and two-dimensional nature contributed to their gradual replacement [7,20]. Bone scintigraphy, which utilizes radioactive tracers to visualize bone activity, offers insights into suture metabolism and growth patterns. Despite being useful, its more invasive nature and radiation exposure limit its long-term application in the pediatric population [21,22]. With advancements in imaging technology, these methods have largely been supplanted by CT and MRI, which offer more detailed and safer evaluations in CRS patients. US, although frequently performed in patients with suspected premature suture fusion, has emerged as a valuable tool for the initial screening and stratification of patients who are worthy of undergoing in-depth instrumental examination.

The main concern when defining the optimal technique between CT and MRI for children undergoing neuroradiological evaluation for CRS (thus, usually requiring iterative monitoring and long-term follow-up) is repeated exposure to ionizing radiation over time [23,24]. Radiation sparing for children affected by CRS is of paramount importance due to the potential long-term risks connected to such repeated exposure to ionizing radiation. Indeed, children are more sensitive to radiation than adults are, and the cumulative effects of multiple imaging procedures can increase the risk of radiation-induced disorders. Traditional CT scans provide detailed images that are crucial for surgical planning and posttreatment evaluation, even at the cost of exposure to significant levels of radiation. Recent advancements in imaging technologies (such as low-dose CT protocols and the increasing resort to MRI examination) offer promising alternatives, minimizing radiation exposure while maintaining diagnostic accuracy. MRI provides the possibility of avoiding ionizing radiation and offers detailed information on cranial plus brain structures at one time. Although MRI has limitations that will be discussed further below, including longer scan times and the need for sedation in younger patients, it represents a safer option when repeated imaging must be performed. Adopting radiation-sparing techniques not only reduces the immediate risks associated with radiation exposure but also aligns with the principle of “as low as reasonably achievable” (ALARA) in pediatric care. Thus, prioritizing these methods ensures that children with CRS receive the necessary diagnostic evaluations with minimal long-term health risks [23,25]. The major advantages and disadvantages of the most relevant neuroimaging techniques for diagnosis, pre-operative surgical planning, and post-operative monitoring of patients with non-syndromic craniosynostosis are summarized in Table 2.

### 3.1. Ultrasound

**Figure 8:**
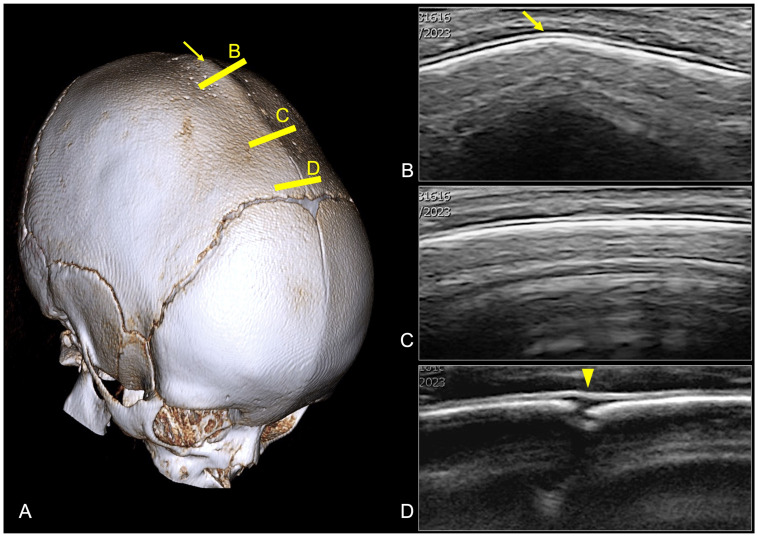
Premature fusion of the sagittal suture in a 12-month-old boy. (**A**) CT volume rendering showing the dolichocephalic shape of the head, with complete fusion of the sagittal suture and sagittal ridge on the medial aspect of the suture. (**B**,**C**) US images obtained at the level of the yellow lines positioned on the 3D CT image showing the fused sagittal suture as a continuous hyperechoic calvaria with loss of the expected hypoechoic gap and the presence of a sagittal ridge (yellow arrow). (**D**) A small portion of the patient’s sagittal suture is shown on the very anterior aspect of the suture close to the anterior fontanelle as a hypoechoic gap (yellow arrowhead).

US has emerged as a noninvasive first-level imaging technique for studying sutures and fontanelles in infants with suspected CRS [26]. Indeed, in the context of CRS, US is particularly useful due to the unique anatomical features of infants, such as open fontanelles and thin skull bones, which allow for an adequate transmission of sound waves. US is adept at evaluating the patency of cranial sutures and fontanelles. By providing real-time imaging, it can reveal whether a suture is prematurely fused or remains open. Normal sutures appear as hypoechoic (black) lines, while fused sutures exhibit an echogenic (white) appearance with loss of the normal suture line [26,27] (Figure 8). The fontanelles, when their patency is preserved, represent the acoustic windows for the visualization of underlying brain structures. The advantages of US include a safe profile due to the absence of ionizing radiation (particularly beneficial for young children who may require multiple imaging studies), the possibility of obtaining dynamic real-time images for immediate assessment and diagnosis, portability, and easy accessibility. Moreover, US is relatively inexpensive compared to other imaging modalities, which makes it a cost-effective option for both initial screening and follow-up studies [28]. Despite its advantages, US has several limitations, including operator dependency (the imaging accuracy is highly dependent on the operator’s skill and experience, and inexperienced operators may miss subtle signs of suture fusion), a restricted field of view compared to CT or MRI (which can make it challenging to assess the entire cranial structure comprehensively, especially in children with complex CRS), and the influence of bone thickening for older infants and toddlers [29]. Therefore, to overcome its limitations, US is often used in conjunction with other imaging modalities. In the near future, technological advancements (such as 3D-US, high-resolution probes, and artificial intelligence) hold promise for enhancing US’s diagnostic reliability and expanding its applications.

### 3.2. Computed Tomography

**Figure 9:**
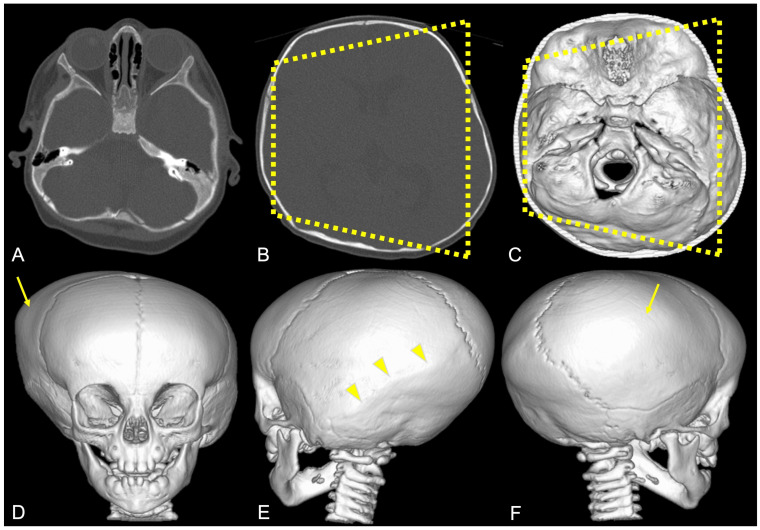
Example of high-resolution three-dimensional CT images (**A**,**B**) of the skull with volumetric image reconstruction (**C**–**F**) in a patient diagnosed with posterior plagiocephaly due to right-sided lambdoid synostosis (yellow arrowheads), with a trapezoidal shape of the head (dotted yellow line) and compensatory left-sided frontal–parietal bossing (yellow arrow).

**Figure 10:**
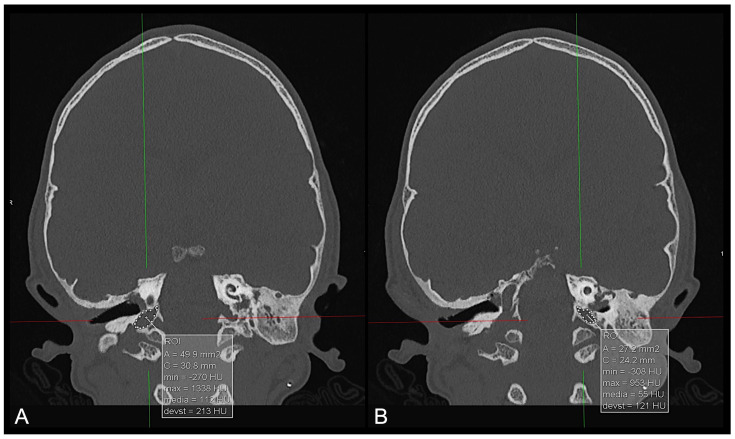
CT multiplanar coronal reconstruction of the jugular foramina in a patient diagnosed with brachycephaly showing a larger right jugular foramen ((**A**)—approximately 50 mm^2^) than on the left side ((**B**)—approximately 27 mm^2^) by means of area calculations obtained by placing manual regions of interest (ROIs—white dotted lines).

CT scans have long been considered the gold standard for assessing CRS due to their ability to provide detailed images of cranial sutures and overall skull morphology with reasonable cost-effectiveness [30]. Detailed bone images are obtained from high-resolution three-dimensional images of the skull, allowing for multiplanar and volumetric image reconstruction, thus enabling precise visualization of suture fusion and the extent of cranial deformities (Figure 9). This is crucial for distinguishing CRS from other cranial deformities and for determining the specific type of CRS (i.e., sagittal, coronal, metopic, lambdoid, or complex). CT imaging can also be used to measure intracranial volume (ICV) and detect any changes in the cranial cavity or foramina (Figure 10). This technique helps for the assessment of the severity of CRS, as well as its impact on the ICP and vascular supply. Detailed CT scans are also used for the presurgical planning of corrective procedures, providing essential information on bony structures requiring reconstruction and guiding the optimal surgical approach to minimize risks and maximize aesthetic/functional outcomes. Advanced 3D reconstructions from CT images also allow for the customization of surgical implants and devices, if needed. Postoperative CT can also be used to assess bone repositioning early, suture patency, and the overall shape and symmetry of the skull, as well as to detect possible complications, such as bone resorption or regrowth of fused sutures, thus allowing for timely intervention.

The rapid acquisition time of CT scans is beneficial for minimizing the need for sedation in young children. However, as previously discussed, ionizing radiation associated with CT poses significant risks, particularly for pediatric patients who are more sensitive to radiation and have a longer lifetime span in which radiation-induced damage can develop. Several efforts have been made to minimize radiation exposure while maintaining image quality by using low-dose protocols and advanced imaging software reconstruction tools. Key technical adjustments in low-dose CT scans include reducing the tube voltage to 80–100 kV and optimizing the tube current to approximately 10–50 mA using automatic exposure control; thin slice thickness (1 mm or less) and increased pitch (1.2–1.5) help maintain image quality and reduce scan time. Advanced iterative reconstruction algorithms further enhance image quality and reduce noise, compensating for lower radiation levels. These modifications ensure that low-dose CT scans offer detailed imaging for diagnosing CRS with minimal radiation risk. Studies confirm that these protocols significantly reduce radiation exposure without compromising diagnostic efficacy, balancing safety and clinical accuracy [25,31,32,33].

### 3.3. Magnetic Resonance Imaging

MRI is an effective alternative to CT that avoids radiation exposure and provides excellent soft-tissue contrast [34]. Generally, MRI is not strictly required in patients with isolated nonsyndromic CRS with neither neurological impairment nor elevated ICP. Conversely, an integrated approach based on the combination of 3D CT and MRI is considered mandatory in cases of complex syndromic disorders, the presence of neurological symptoms, or signs of increased ICP [35]. MRI should not be limited to the conventional standard examination but should also include specific sequences for comprehensive bony structures, CSF spaces, brain parenchyma, and pathways of venous outflow assessment. Here, we provide an overview of MRI sequences that can provide major benefits, both in the diagnostic presurgical stage and for postsurgical monitoring, and that have the potential to complement or even supersede CT examination in the evaluation of patients with craniosynostoses.

**Figure 11:**
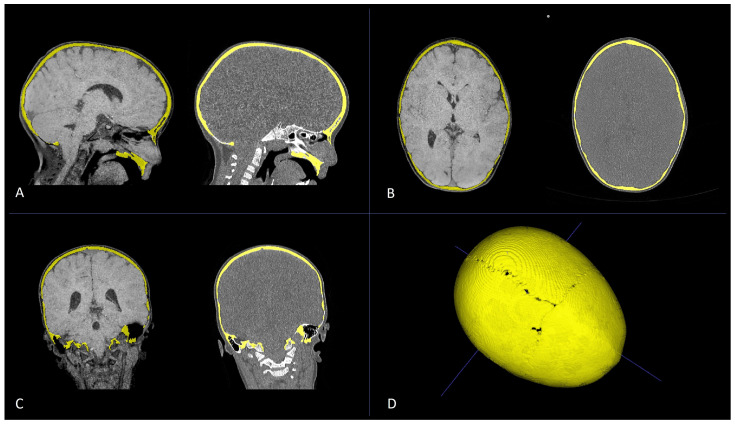
Multiplanar reconstruction (**A**–**C**) of CT scan (**left**) and black-bone MR image (**right**) of a patient diagnosed with PT for premature fusion of the metopic suture. Axial (**A**), sagittal (**B**), and coronal (**C**) plane reconstructions were used to obtain bone segmentation (yellow) and subsequent volumetric rendering from black-bone MR images (**D**).

**Figure 12:**
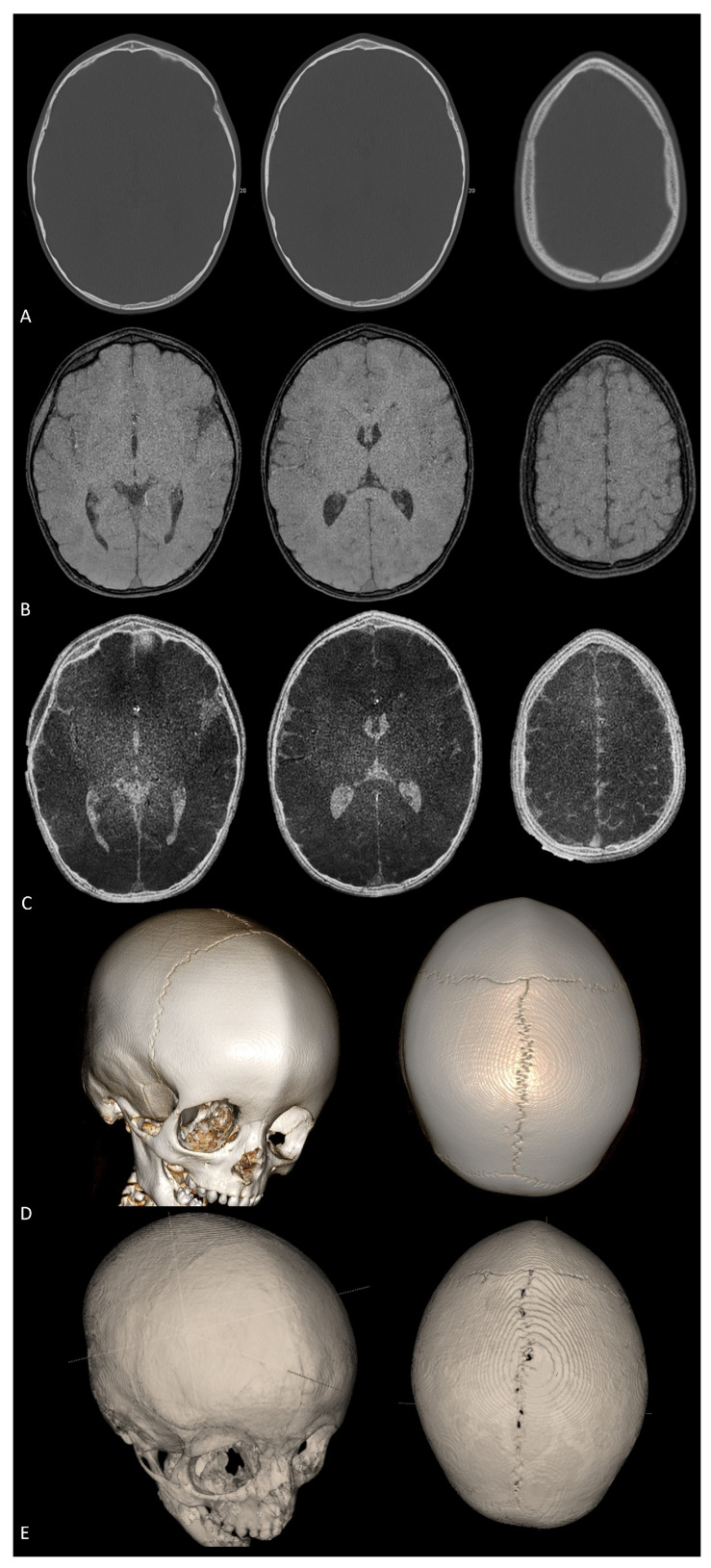
Axial CT scan (**A**), axial black-bone MRI (**B**), and axial black-bone MRI CT-like representation (**C**) at comparable levels in the same patient shown in Figure 11; 3D CT reconstruction (**D**) and 3D black-bone imaging postprocessing reconstruction (**E**) demonstrating comparable visualization of cranial sutures, metopic ridge, and overall skull deformity.

Three-dimensional MRI with CT-like bone contrast, encompassing drawing cortical bone with MRI, is becoming increasingly feasible due to the development of specific techniques that provide CT-like bone contrast images that can complement the assessment of soft tissues within a single MRI examination [36]. In particular, these techniques include black-bone ultrashort echo time (UTE), zero-time echo (ZTE), T1-weighted gradient recalled echo, and susceptibility-weighted imaging (SWI) [37,38,39,40]. Among them, black-bone MRI probably represents the most promising advancement in the imaging of pediatric CRS [41,42]. Black-bone UTE is a gradient-echo MRI sequence with a short echo time, short repetition time, and optimal flip angle that enhances the contrast difference between cortical bone and the adjacent soft tissues. This results in images where bones appear dark (hence the name), providing a clear delineation of the bony anatomy against the surrounding soft tissues and enhancing the visualization of cranial sutures. Due to image postprocessing algorithms, it also allows for CT-like volumetric skull reconstruction comparable to that obtained with CT scans [43,44] (Figure 11 and Figure 12). Its ability to provide detailed bone and soft-tissue images without radiation exposure offers a safer alternative to traditional CT scans, making it ideal for longitudinal studies in CRS pediatric patients [42]. Despite its advantages, black-bone MRI is not yet widely available, particularly in resource-constrained settings. In this regard, the higher cost of black-bone MRI may be a barrier to its widespread adoption. Moreover, this technique is still evolving, and further comparative studies between black-bone MRI, CT, and conventional MRI are needed to validate its effectiveness or establish it as a standard imaging modality for routine clinical use in CRS assessment [45,46]. In particular, scanner and acquisition parameter-related variations may impact image quality, thus increasing the need for standardized protocols and posing challenges for consistent interpretation/routine clinical application. The development of standardized imaging protocols and the training of radiologists in the interpretation of black-bone MR images will enhance its clinical use and ensure image reproducibility. Although challenges related to availability, cost, and standardization remain, the future of black-bone MRI in CRS assessment looks promising, with the potential to become an integral part of pediatric craniofacial imaging both in syndromic and nonsyndromic cases [34].

**Figure 13:**
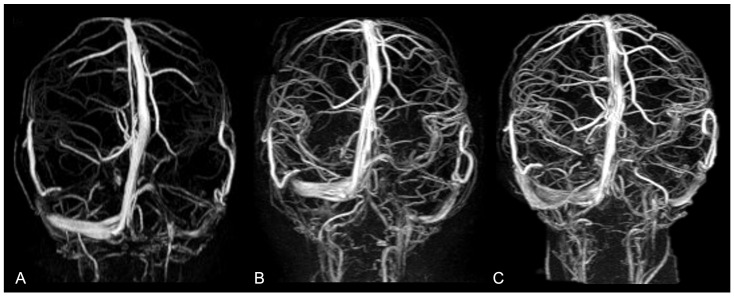
Non-contrast intracranial phase-contrast MR venography in a 28-month-old patient who underwent surgery for nonsyndromic complex CRS (premature fusion of lambdoid sutures, sagittal suture, and inferior aspect of left coronal suture), documenting progressive improvement in the visualization of dural venous sinuses (superior sagittal, transverse, straight, and sigmoid sinuses), larger deep cerebral veins (cavernous sinus), and cortical veins at three different time points: (**A**) before surgery; (**B**) 1 month after surgery; (**C**) 6 months after surgery.

Magnetic Resonance Angiography (MRA) provides high-resolution images of intracranial vasculature without resorting to ionizing radiation or intravenous contrast agents. It ensures a detailed visualization of the arteries and (above all) veins and dural sinuses, thus allowing for a better understanding of vascular anatomy and anomalies related to altered skull growth in craniosynostoses [47]. Indeed, it is well known that CRS can impact venous drainage patterns and that increased dural venous sinus pressure can be commonly observed in noncomplex craniosynostoses. However, it is still unclear whether venous obstruction results from primary bone disorder or whether it occurs at a later stage as a consequence of chronic high ICP in the posterior fossa. The most frequent intracranial venous abnormalities observed in nonsyndromic craniosynostoses include small jugular foramina, an aberrant course of the jugular veins, and extensive transosseous venous collaterals [48,49]. MRA enables an accurate depiction of venous hypertension, major sinus stenosis, and collateral venous pathways (representing compensatory mechanisms for obstructed primary outflow routes). It allows for precise measurement of the size/shape of dural sinuses and (coupled with morphological imaging) drives the identification of the site of venous drainage stenoses, therefore providing critical information for surgical planning. MRA is also used for assessing vascular changes after surgery, documenting venous hypertension resolution and normal venous drainage restoration, and detecting late-onset complications or changes potentially requiring reintervention [50] (Figure 13).

**Figure 14:**
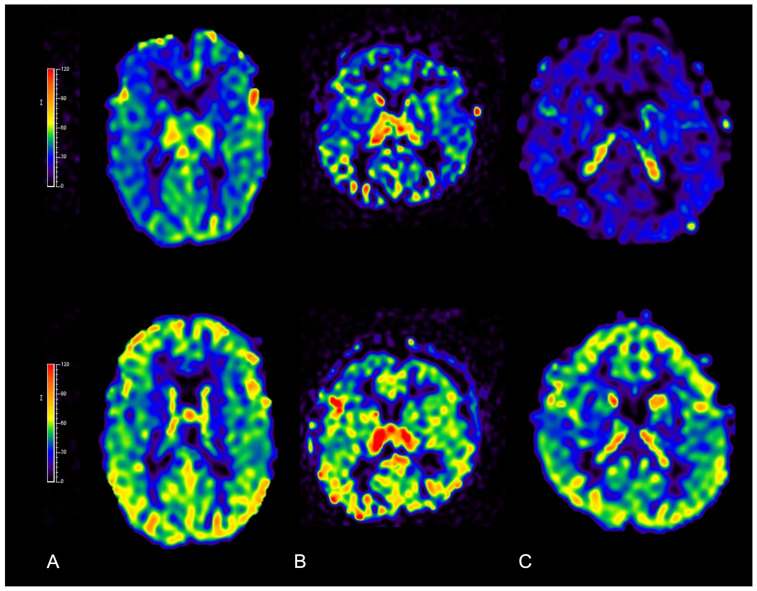
Qualitative increase in cerebral blood flow demonstrated with ASL perfusion MRI before (**upper row**) and 6 months after surgery (**bottom row**) in three patients diagnosed with nonsyndromic isolated CRS: (**A**) scaphocephaly; (**B**) anterior plagiocephaly; and (**C**) trigonocephaly. Threshold color scale bars are shown on the left.

Perfusion MRI: Premature fusion of skull sutures also influences brain perfusion in patients with isolated nonsyndromic CRS, with potential functional repercussions that go far beyond pure cosmetic effects. Among perfusion MRI techniques, arterial spin labeling (ASL) provides a noninvasive cerebral blood flow qualitative/quantitative evaluation by magnetically labeling arterial blood water as it flows into the brain without requiring intravenous contrast-media administration. This method has become increasingly important for the evaluation and management of craniosynostosis hemodynamic changes [51]. CRS often results in regional variations in brain perfusion due to abnormal skull growth and altered venous drainage. At baseline, ASL can detect the presence or absence of regional perfusion differences [52,53], helping to identify areas at risk of hypoperfusion, which may contribute to neurodevelopmental issues. Then, following cranial vault remodeling or other corrective surgical approaches, ASL can be used to document improvements in cerebral perfusion and to identify areas of persistent reduced perfusion at long-term follow-up [54,55] (Figure 14). ASL has also shed new light on the pathophysiology of CRS, opening new avenues for research on how altered skull growth affects brain hemodynamics and development.

**Figure 15:**
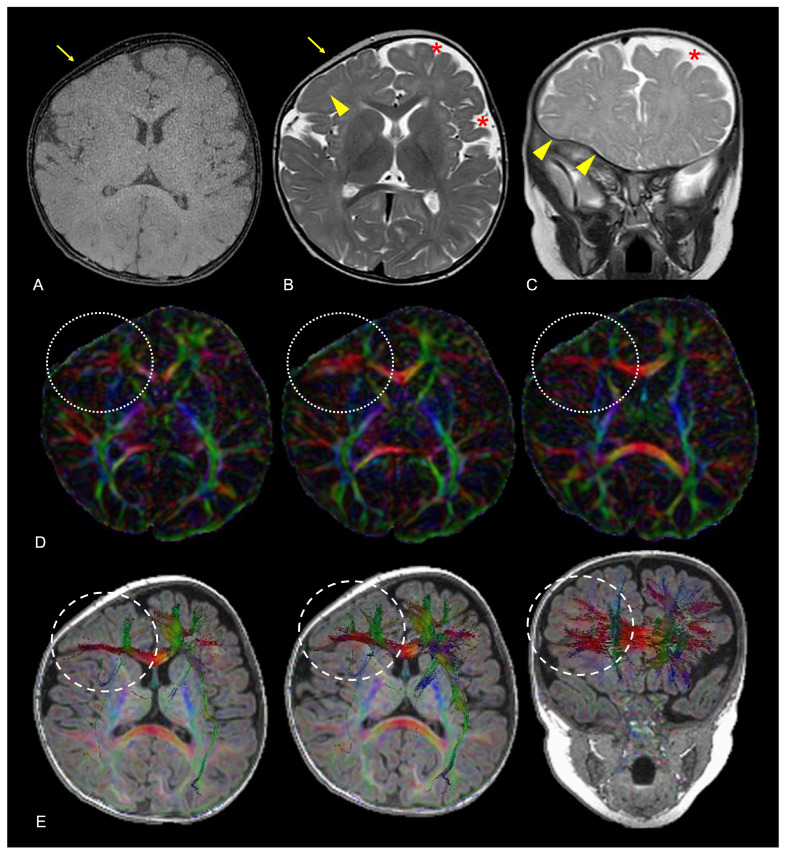
Axial black-bone MR image (**A**), axial TSE T2W image (**B**) and coronal TSE T2W image (**C**) of a 9-month-old female patient diagnosed with anterior plagiocephaly due to premature fusion of the right coronal suture (yellow arrows); aberrant adaptation of the right frontal lobe nervous tissue is indirectly demonstrated by the anomalous orientation of the frontal and frontal-insular sulci both on the axial and coronal planes (yellow arrowheads) compared to the left side, coupled to the asymmetric representation of adjacent CSF spaces (red asterisks). Axial FA maps (**D**) and color-coded representation of diffusion tensors on axial and coronal reconstructions superimposed on 3D-T1W images (**E**) confirmed altered fractional anisotropy (white dotted lines) and aberrant white matter fiber orientation (white dashed lines) due to white matter structural adaptive changes in the corresponding area. Color legend: red for left–right; blue for superior–inferior; and green for anterior–posterior.

Tractography and functional magnetic resonance imaging (fMRI) are advanced neuroimaging techniques, such as fractional anisotropy (FA) and radial diffusivity measurement, tractography reconstruction, and functional magnetic resonance imaging (MRI), and are gaining more ground in CRS-related brain change assessment, as they provide detailed insights into brain connectivity. FA maps and tractography derived from diffusion tensor imaging (DTI) help visualize and localize potential disruptions in white-matter pathways secondary to premature suture fusion (Figure 15). Tractography can also help surgeons avoid critical bundles during cranial vault remodeling, minimizing the risk of postoperative neurological deficits [55]. It can also be used to monitor changes in white-matter integrity following surgery or identify areas that may require further clinical attention. fMRI measures brain activity by detecting changes in blood flow and oxygen extraction, providing insights into the functional organization of the brain. In CRS, fMRI is used for several purposes, including identifying functional regions impacted by altered skull growth and subsequent brain compression, planning surgery to ensure functional area preservation, and assessing neuroplasticity following surgery, for a better understanding of how the brain compensates for any structural change, and potentially planning specific rehabilitation strategies [56]. The combined use of tractography and fMRI offers a holistic assessment of how CRS affects the brain, considering both the physical disruption of white-matter tracts and the functional implications for brain activity. These data may be used not only for tailored therapeutic approaches intended to improve overall patient outcomes but also for research purposes oriented toward a better comprehensive understanding of CRS-related brain changes.

### 3.4. Digital Subtraction Angiography (DSA)

DSA, although representing the gold standard in the diagnosis of vascular abnormalities, has a very limited role in the treatment of nonsyndromic craniosynostoses. Its application is limited to those cases in which significant vascular alterations have been identified on CT and/or MRI. Rather than for vascular lesion demonstration or depiction, DSA may prove useful when an endovascular treatment of important venous abnormalities is required before surgery to reduce the expected bleeding.

## 4. Associated Structural Brain Abnormalities: Beyond Sutures and Fontanelles

Unlike syndromic craniosynostoses, which are associated with pervasive congenital anomalies (frequently multisystemic) and genetic conditions, nonsyndromic CRS usually occurs in isolation, frequently with no identifiable putative genetic cause. For a long time, nonsyndromic isolated CRS has eminently been viewed as a purely aesthetic issue. Therefore, the main goal in treating this craniofacial condition is to correct the premature fusion of skull sutures to allow for normal brain and skull growth, which involves releasing the fused sutures and reshaping the skull to achieve a more typical head shape. However, morphological changes in the skull can still profoundly influence the developing brain [57]. Fused sutures limit skull growth near the suture, and adjacent structures compensate by adapting to this impairment and growing in other areas to allow for brain development. Indeed, the current research shows that children with nonsyndromic CRS have a greater incidence of below-average scores than those with normal sutures, suggesting an increased risk of neurodevelopmental issues and cognitive impairment [58]. Moreover, because the growth of the skull base is closely coordinated with the growth of the cranial vault, premature suture fusion in nonsyndromic CRS may lead to a tilted and uneven cranial base that may influence the normal development of the posterior cranial fossa, therefore impacting the cerebellum and brainstem [3]. Neuroimaging techniques, with specific reference to MRI, can provide an accurate depiction of possible intracranial and spinal associations and (by means of advanced MRI sequences) can also allow for a deeper understanding of subtle alterations in nervous tissue organization that may not be visible with conventional imaging.

### 4.1. Intracranial Volume and Intracranial Pressure Alterations

Premature suture fusion can lead to increased ICP due to abnormal skull growth with impaired ICV. In most children with CRS, the ICV is typically within normal ranges or tends to reach normal volumes within the first six months of life. These findings suggest that, despite the limitations ascribable to prematurely fused sutures, the skull compensates through growth at unaffected suture sites, thereby maintaining a normal ICV during development [59]. Conversely, the prevalence of increased ICP in pediatric patients with single-suture nonsyndromic CRS is more controversial, although several studies have reported direct or indirect signs of increased ICP of greater or lesser duration in these patients since the early stages of life [60,61,62,63,64,65,66]. At present, the incidence of increased ICP in patients with single-suture CRS ranges from 15% to 20%. The frequency and severity of increased ICP in patients with nonsyndromic CRS reflects the severity and precocity of TS, as well as its progression over time [61,62,63,64,65,66,67,68,69]. Although isolated nonsyndromic CRS usually does not lead to hydrocephalus and has a prevalence of cerebral ventricle dilation comparable to that observed in the normal population, it can still result in increased ICP [70]. The pathophysiology of increased ICP in those patients is probably explained by the combination of hydrovenous, deformative, and malformative causes. Craniocerebral disproportion with ICV-normal skull deformity but altered brain/CSF volume distribution, which is variably coupled to impaired venous drainage and posterior fossa abnormalities, may be responsible for a prolonged or transient increase in ICP. As previously described, neuroimaging can help in assessing the relationship between nervous tissue, CSF, and cranial volume through the use of volumetric acquisitions. It may also allow for the assessment of hydrovenous disproportion due to the use of perfusion and angiographic techniques. Elevated ICP can compress brain structures, potentially causing ventricular enlargement and affecting cerebral perfusion. Increased ICP may lead to symptoms such as headaches, visual disturbances, and (in more severe cases) developmental delays and cognitive deficits. Early detection and intervention are crucial for mitigating these risks [47].

### 4.2. Possible Associations with Macroscopic Alterations in Brain Morphology

**Figure 16:**
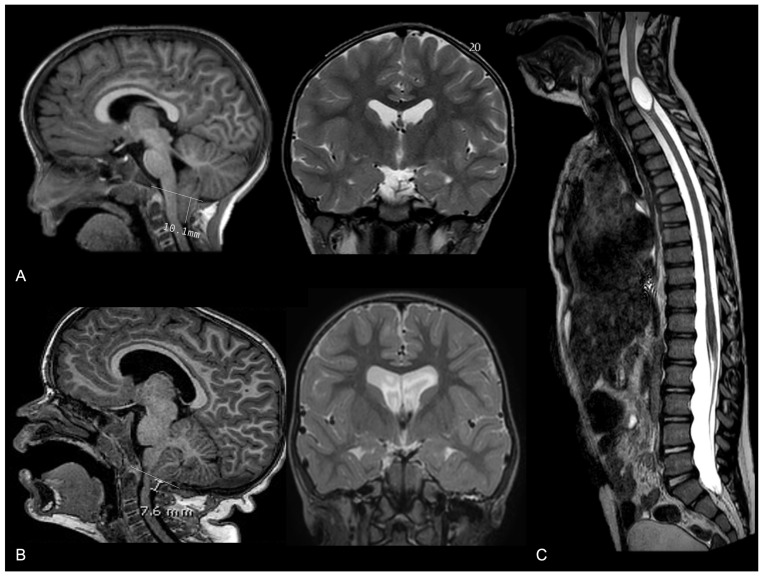
Three cases of nonsyndromic trigonocephaly associated with (**A**) isolated tonsillar herniation with a normal ventricle size; (**B**) tonsillar herniation coupled to moderate supratentorial ventricular dilation; and (**C**) cervical spine syringomyelia.

When a cranial suture fuses prematurely, it restricts the growth of the skull in a perpendicular direction to the suture. Consequently, the brain and skull expand in directions where the sutures remain open, leading to characteristic skull shapes. This compensatory growth can lead to abnormal brain shapes, with elongated or distorted brain hemispheres. In conditions such as unilateral coronal or lambdoid synostosis, the asymmetrical fusion of sutures results in a significant asymmetry in both the skull and brain structures. The affected side may show reduced growth, while the opposite side compensates. Moreover, the growth of the skull base occurs in close coordination and symmetry with the growth of the cranial vault. Therefore, premature suture fusion may lead to a tilted and uneven cranial base, which may influence the normal development of the posterior cranial fossa housing the cerebellum and brainstem, which (coupled with the altered cranial volume and altered intracranial dynamics) can exacerbate neurological manifestations. However, the involvement of skull base synchondroses is rare in single-suture CRS, with the two main potential exceptions being plagiocephaly and trigonocephaly, in which cranial base deformities are more frequently encountered [7]. One of the most common posterior fossa malformations linked to CRS is Chiari malformation type 1 (CM1), where cerebellar tonsils extend into the spinal canal. Recent studies have described a prevalence of 8% CM1 in patients with CRS, with higher percentages of specific subtypes, such as isolated lambdoid synostosis and complex nonsyndromic CRS [71]. The descent of cerebellar tonsils below the foramen magnum in those patients can disrupt CSF hemodynamics, leading to further manifestations such as syringomyelia or ventricular dilation (Figure 16). Syringomyelia, characterized by the development of a fluid-filled cyst within the spinal cord, occurs in a minority of CRS patients and is usually associated with a more severe neurological phenotype. Management of CM1 in the context of CRS often involves a surgical intervention to decompress the foramen magnum and restore normal CSF flow; this approach can alleviate symptoms of ventricular dilation and prevent the progression of syringomyelia [72,73]. Conversely, an isolated hydrocephalus is considered very rare in these patients, and in rare cases where it does occur, it can generally be explained by another nonsynostosis-related underlying condition. Overall, the association between isolated CRS and CM1, along with the potential development of ventricular dilation or syringomyelia, further highlights the importance of comprehensive neuroimaging and vigilant clinical follow-up in these patients.

### 4.3. Microstructural Abnormalities and Possible Impacts on Brain Development

All the abovementioned asymmetries and compensatory mechanisms can influence brain connectivity and may be associated with functional deficits, such as motor or cognitive impairments, although many children adapt well and develop normally. Therefore, the extent and nature of this impact vary widely among individuals, and advanced MRI techniques may shed new light on the early identification of the features suggestive of neurodevelopmental and cognitive issues (thus allowing for better patient stratification due to early diagnosis). Recent research indicates that, compared with the general population, children with nonsyndromic CRS have an increased risk of neurodevelopmental issues. These issues may include language delays, learning disabilities, and behavioral problems. The type and severity of such disorders often correlate with the number of sutures involved and the presence of elevated ICP. Generally, children with single-suture CRS tend to have better outcomes than those with multiple sutures involved [58]. Such reasoning also applies to cognitive function. Indeed, the level of cognitive development in children with CRS can vary, and while many patients exhibit normal cognitive functioning, some of them may experience difficulties (particularly in visual–spatial skills, executive function, and attention). Recent statistics show that neurocognitive delay can be observed in up to 50% of school-aged children diagnosed with this condition [57,74]. Advanced MRI techniques, such as fMRI and tractography, may prove the presence of some abnormalities in structural and functional connectivity [56,75,76,77], highlighting network alterations that could affect normal executive functioning the most. With this background, comprehensive MRI examination coupled with early neuropsychological assessment may be adopted in nonsyndromic CRS patients to identify at-risk phenotypes in a timely manner and to monitor the effect of therapeutic interventions over time.

## 5. Postoperative Imaging Challenges

Generally, nonsyndromic craniosynostoses require a single intervention to achieve head-shape correction. However, concerning the time of surgical correction, there is a certain variation in age for intervention, largely depending upon the type/extent of synostosis, the envisaged surgical procedure, and the presence of associated abnormalities. In more complex cases, the surgical treatment must be more flexible and solution-oriented in order to minimize the need for re-intervention [78,79,80]. With this in mind, it becomes clear how peri-operative neuroimaging choice and timing should be driven by the same principles, and non-ionizing techniques should be preferred whenever possible.

While pre-operative image acquisition is modeled on the ideal age for intervention, post-operative image acquisition is more variable [78]. Usually, early post-operative examinations should be carried out (when necessary) within a few hours/days from intervention to detect early complications. Conversely, medium–long-term neuroimaging monitoring should be carried out at around 6 months from the surgical procedure to highlight salient changes in head shape and intra-cranial findings. Indeed, postoperative imaging plays a crucial role in assessing the success of surgical procedures in patients with nonsyndromic craniosynostoses, documenting bone healing and detecting any early or late complications. However, imaging monitoring presents some major challenges that radiologists must navigate to ensure optimal patient management.

Regarding image quality and artifact interference, neurosurgical interventions sometimes involve the use of implants, plates, screws, or external osteodistraction. These materials, although safe, can be responsible for artifacts in imaging studies, particularly in CT and MRI scans, masquerading critical details and complicating the assessment of bone healing and implant positioning. Overcoming metallic artifacts in the postoperative brain imaging of patients with neurosurgical implants presents a significant challenge, but several strategies can help mitigate these distortions and enhance image quality. With respect to CT scans, imaging parameter adjustments (such as optimizing slice thickness and orientation) can help reduce the impact of metal implants or other devices and limit the extent of related artifacts. Modifying parameters, such as the kilovoltage peak and milliamperes, can also influence artifact reduction. When available, dual-energy CT (DECT) scans can better distinguish between metal and surrounding tissues by using two different energy levels. DECT enhances the ability to visualize both soft tissues and bone structures around implants. Image-quality preservation can also be achieved by means of iterative reconstruction algorithms and postprocessing software, which enhance image quality by compensating for the distortions introduced by metal implants [81,82]. Concerning MRI acquisition, specific MRI sequences increasingly available from most scanners and vendors (and globally known as metal artifact reduction sequences or MARS) can be adopted to reduce such interference [83]. Signal loss due to static dephasing can be largely corrected by using spin–echo sequences, while certain distortions can be minimized by selecting the appropriate scanning parameters. Fat-suppression issues can be addressed with Dixon techniques or short-time inversion recovery imaging, despite the lower signal-to-noise ratio. Geometric distortion artifacts can be corrected through various methods. View-angle tilting effectively handles in-plane displacement artifacts, but a more comprehensive correction can be achieved using multispectral imaging methods, although with longer scan times. Understanding the causes of metal-induced artifacts and selecting the appropriate correction technique are crucial for minimizing artifacts in specific applications. When both CT and MR images are heavily compromised by artifacts, US and other nonradiative techniques can alternatively be employed [29]. By integrating different and complementary techniques, significant information can generally be collected, and the overall reliability of postoperative brain imaging in CRS patients with neurosurgical implants has improved.

For the assessment of bone healing and fusion, an accurate assessment of bone healing and regrowth at the surgical site is crucial. Differentiating between normal bone remodeling and the pathological refusion of cranial sutures can be challenging, especially in the early postoperative period [84,85]. Several methods for evaluating the pathological refusion of cranial sutures during postoperative CRS monitoring have been developed. At clinical examination, asymmetrical skull growth, raised ridges along sutures, and persistent or recurrent cranial deformities are key indicators of surgical failure. Persistent headaches, developmental delays, and behavioral changes are also suggestive of refusion. In such cases, imaging is crucial, with CT scans revealing premature suture fusion and bone bridging and MRI showing associated brain anomalies and ICP changes. US may be effective for detecting early signs of refusion in infants, but it often proves to be far less effective in toddlers and children.

As to identifying early postsurgical complications, detecting early complications such as infection, bone resorption, and intracranial hypertension is pivotal for prompt intervention and improvement in patients. These complications can be difficult to detect at a very initial stage, necessitating a high level of expertise and vigilance in interpreting postoperative images. MRI is often superior to CT in the early identification of early complications and potentially life-threatening conditions. In some cases, such as postoperative infections or vascular complications, the use of contrast media may be needed.

Overall, long-term follow-up multimodal imaging is necessary to monitor the growth and development of the cranial vault in these patients. This requires a consistent imaging protocol and the ability to compare images over time, which can be complicated by variations in imaging techniques and equipment. This is all the more true if considering MRI examination, whose reproducibility is particularly affected by variations in scanner vendors, field strength, coils, and sequence parameters.

## 6. Conclusions

Nonsyndromic CRS still represents an active research field since not all evolutionary and morphostructural aspects related to isolated premature suture fusion have been elucidated to date. Current studies have examined not only the morphological and cosmetic impacts of these synostoses but also their neurocognitive effects, and many affected children have experienced intracranial hypertension and brain morphology changes since early infancy. These patients often exhibit language, attention, visual–spatial, and cognitive processing deficiencies that may not be resolved by surgery and that cannot be fully explained by the cranial vault deformity itself, some of which do not completely revert even after corrective surgery. Despite these considerations, surgery is still the principal therapeutic option, with early intervention (before one year of age) being preferable. Future research should aim to better understand the link between synostosis and cognitive impairment and explore the genetic mutations involved. From a diagnostic standpoint, while CT scans remain the gold standard for identifying nonsyndromic craniosynostoses due to their speed and excellent visualization of bony structures, other techniques are gaining ground to avoid ionizing radiation. Although ionizing-radiation-free methods (such as US and MRI) are increasingly being used in this specific clinical setting, to date, they have only partially reached the availability, reproducibility, and diagnostic consistency of CT scans in daily clinical practice. However, it has become clear that the implementation of advanced MRI techniques as a reference diagnostic tool for CRS assessment could help in providing new insights and offer new perspectives for a better multidimensional understanding and management of the disease.

## Figures and Tables

**Table 1:** Physiologic timeline of major cranial vault suture fusion.

Major Sutures	Complete Fusion	Details
Metopic suture	3–9 months	Fuses up to 6 years or never closes
		Closes from nasion to anterior fontanelle
Sagittal suture	21–30 years	Unfused through childhood
		Narrows from the 1st month
Coronal suture	~24 years	Unfused through childhood
		Narrows during childhood
Lambdoid suture	~26 years	Unfused through childhood
		Common site of Wormian bones

**Table 2:** Table summarizing major advantages and disadvantages of the most relevant neuroimaging techniques for diagnosis, pre-operative surgical planning, and post-operative monitoring of patients with non-syndromic craniosynostosis.

Neuroimaging	Advantages	Disadvantages
Ultrasound	Non-irradiating technique Fast and repeatable Non-invasive No sedation required Widely available Low-cost	User-dependent Inconclusive if poor patients’cooperation Limited use in relatively advanced-age patients Poor evaluation of deeper structures
Computed tomography	Fast acquisition Readily accessible Sedation generally not required Widely available Limited cost Excellent bone evaluation Multiplanar and 3D reconstructions easily available	Irradiating technique (thus repeated CT scans over time should be limited) Limited evaluation of soft tissues and associated brain abnormalities Iodinated contrast media required for vascular structures depiction
Magnetic resonance imaging	Non-irradiating technique Repeatable over time Excellent soft tissue definition Excellent potential due to advanced techniques Gadolinium-based contrast media generally not required Reference method for associated brain and spine abnormalities search	Sedation usually required Not always readily accessible Long acquisition Higher cost Specific professional experience required for data interpretation Longer data post-processing
Digital subtraction angiography	Excellent vascular structures depiction Real-time observation of blood flow dynamics Potentially combinable with interventional procedures, when required (cost-effective)	Irradiating technique More invasive Specific professional experience required Iodinated contrast media always required Sedation usually required Higher cost Not always readily accessible Limited spatial resolution Very poor or no evaluation of structures other than vascular

## Data Availability

No new data were created or analyzed in this study. Data sharing is not applicable to this article.

## Abbreviations

ALARA = as low as reasonably achievable; ASL = arterial spin labeling; CM1 = Chiari malformation type 1; CRS = craniosynostoses; CSF = cerebrospinal fluid; CT = computed tomography; DECT = dual-energy CT; DSA = digital subtraction angiography; DTI = diffusion tensor imaging; FA = fractional anisotropy; fMRI = functional MRI; ICP = increased intracranial pressure; ICV = intracranial volume; MRI = magnetic resonance imaging; SWI = susceptibility-weighted imaging; US = ultrasound; UTE = ultrashort echo time; ZTE = zero-time echo.
